# Nutraceuticals and Herbal Food Supplements for Weight Loss: Is There a Prebiotic Role in the Mechanism of Action?

**DOI:** 10.3390/microorganisms9122427

**Published:** 2021-11-25

**Authors:** Alexander Bertuccioli, Marco Cardinali, Marco Biagi, Sara Moricoli, Ilaria Morganti, Giordano Bruno Zonzini, Giovanna Rigillo

**Affiliations:** 1 Department of Biomolecular Sciences, University of Urbino Carlo Bo, 61029 Urbino, Italy; 2 Department of Internal Medicine, Infermi Hospital, AUSL Romagna, 47900 Rimini, Italy; marco.cardinali@auslromagna.it; 3 Department of Physical Sciences, Earth and Environment, University of Siena, 53100 Siena, Italy; marco.biagi@unisi.it; 4 AIFeM, 48100 Ravenna, Italy; sara.moricoli@libero.it (S.M.); ilariamorganti93@gmail.com (I.M.); giordanozonzini@gmail.com (G.B.Z.); 5 Department of Life Sciences, University of Modena and Reggio Emilia, 41125 Modena, Italy; giovanna.rigillo@unimore.it

**Keywords:** microbiota, prebiotic, weight loss, food supplements, botanicals

## Abstract

Numerous nutraceuticals and botanical food supplements are used with the intention of modulating body weight. A recent review examined the main food supplements used in weight loss, dividing them according to the main effects for which they were investigated. The direct or indirect effects exerted on the intestinal microbiota can also contribute to the effectiveness of these substances. The aim of this review is to evaluate whether any prebiotic effects, which could help to explain their efficacy or ineffectiveness, are documented in the recent literature for the main nutraceuticals and herbal food supplements used for weight loss management. Several prebiotic effects have been reported for various nutraceutical substances, which have shown activity on *Bifidobacterium* spp., *Lactobacillus* spp., *Akkermansia muciniphila*, *Faecalibacterium prausnitzi*, *Roseburia* spp., and the Firmicutes/Bacteroidetes ratio. Different prebiotics have beneficial effects on weight and the related metabolic profile, in some cases even acting on the microbiota with mechanisms that are completely independent from those nutraceuticals for which certain products are normally used. Further studies are necessary to clarify the different levels at which a nutraceutical substance can exert its action.

## 1. Introduction

Overweight and obesity are related pathological conditions with a significant impact at the cardiovascular, metabolic, musculoskeletal, and oncological levels, representing a significant global public health problem [1]. The traditionally proposed therapeutic approaches act at the nutritional, psychological, lifestyle (abolishing sedentary lifestyle and promoting physical exercise), and pharmacological levels, in combination with the consumption of food supplements [2]. Watanabe et al. recently identified and examined 33 food supplements most used for weight loss, dividing them according to the main effect and depending on the primary impact on nutrient absorption, appetite regulation, energy expenditure modulation, and fat and carbohydrate metabolism [2]. Substances considered prebiotic, which specifically stimulate components of the microbiota capable of providing positive effects, such as increasing immune function and protection from pathogens, improve host metabolism and nutrient absorption [3]. This can have a significant impact on the metabolism of various substances used as food supplements. Based on these aspects, the aim of this review is to evaluate the role of prebiotics in the weight loss activity of the main natural products identified, which has been increasingly investigated and reported recently in the scientific literature.

## 2. Materials and Methods

The literature search was carried out until July 2021. MEDLINE, Cochrane library, and Google Scholar were consulted using the following keywords: “nutraceuticals” or “herbal food supplements” or “natural products” and “weight loss” and “prebiotic”, “microbiota”, “metagenomic”, and “gut flora”. The analysis included data derived from the following types of studies: prospective observational studies, retrospective studies, and randomized clinical studies. In vitro and in vivo studies were only taken into account regarding substances investigated in clinical trials.

## 3. Results

The retrieved data indicated that many different food supplements, both herbal and not, have been investigated in humans for their prebiotic effect and their capacity to modulate gut microbiota. (Table 1). In detail, we found robust data for the following:

1. Supplements with the main goal of nutrient absorption (green tea, ginseng, chitosan, β-glucans, psyllium, guar gum, and inulin);
2. Supplements with the main goal of appetite regulation (whey proteins and chlorogenic acid);
3. Supplements with the main goal of energy expenditure modulation (curcumin and L-carnitine);
4. Supplements with the main goal of fat metabolism (resveratrol and flaxseed).

Moreover, for each substance, we reported data related to effects on weight loss through the microbiota modulation in animal models, considering that results obtained in animal models do not always reflect observations in humans.

### 3.1. Food Supplements with the Main Goal of Nutrient Absorption

#### 3.1.1. Green Tea

Green tea (*Camellia sinensis* Kuntze) is a herb whose preparations have been extensively used in the traditional Chinese medicinal system. It possesses several positive health effects due to polyphenols, which are its most bioactive compounds that have demonstrated many properties, such as antioxidant, antiaging, and anti-inflammatory action [4]. In humans, using 16S rRNA sequencing, it was found that green tea consumption increased the relative abundance of Firmicutes and Actinobacteria, and decreased the concentration of phylum Bacteroidetes. In addition, short chain fatty acid (SCFA)-producing bacteria, such as *Roseburia* spp., *Faecalibacterium* spp., *Eubacterium rectale* group, *Blautia* spp., *Coprococcus* spp., and *Bifidobacterium longum*, were increased by green tea, while other species within the genus *Prevotella* were decreased [5]. Barcena et al. observed that green tea polyphenols were able to increase the concentration of *Faecalibacterium prausnitzii* in a mouse model. The modulation of this gut microbiota species seems to be correlated with the alleviation of high fat diet (HFD)-induced weight gain and associated with intestinal and liver inflammatory response in HFD mice [6,7]. In HFD-fed mice supplemented with green tea extract, an increase in Bacteroidetes and *Oscillospira* spp. families and a reduction in Peptostreptococcaceae concentration was observed. Additionally, the Firmicutes/Bacteroidetes ratio was reduced by green tea polyphenols and *Akkermansia muciniphila* was positively regulated [8,9]. Many in vitro studies have examined the plausible prebiotic role exerted by green tea; in particular, the ability of green tea polyphenols to modulate gut microbiota has been demonstrated. Investigations on Caco2 intestinal cells showed that the abundance of some pathogenic bacteria, such as *Clostridium perfringens*, *Clostridium difficile*, and *Bacteroides* spp., was significantly lower, while the levels of commensal bacteria, such as *Lactobacillus* and *Bifidobacterium* spp., were increased by green tea polyphenols [10,11]. Considering the evidence, green tea may have a potential antiobesity activity by modulating gut microbiota.

#### 3.1.2. Ginseng

Ginseng consists of the dried roots of *Panax ginseng* C.A. Meyer. The main phytochemicals compounds of ginseng are triterpenic saponins, called ginsenosides and polysaccharides. The most well-known is Asian ginseng. On the other hand, *Panax quinquefolius* L. is better known as American ginseng; it mainly differs from Asian ginseng for the quali-quantitative profile of different ginsenosides [12]. Both American and Asian ginseng have been investigated in animal models and in clinical trials for their role in weight loss and gluco-lipidemic profile improvement [13]. Even most clinical trials fail to address a clear effectiveness of ginseng due to the variability of administered preparations, herbal combinations, and dosages [14]; recent investigations have remarked the high potential of ginseng in body weight control through the modulation of gut microbiota. A clinical exploratory study that enrolled 10 obese women aged 40–60 years showed that 8 g/day of a chemically characterized *P. ginseng* extract administered for 8 weeks exerted a weight loss effect and modest modifications of gut microbiota species compared to untreated healthy obese participants. The antiobesity effectiveness of the treatment was related to the different gut microbiota structure before the administration of ginseng. Differences in bacterial communities before and after ginseng administration in both groups exhibited how Tenericutes, Bacteroidetes, and Firmicutes were more represented in women who underwent a more evident biological effect prior to ginseng administration. The relative composition of Actinobacteria and Proteobacteria showed significantly lower abundances [15]. In an in vivo study, the aqueous extracts of two different Asian ginseng preparations, white and red ginseng (oven dried and steam processed and then oven dried, respectively) were administered to obese mice for 10 weeks. Both white and red ginseng were found to be effective as antiobesity agents, but white ginseng, characterized by a lower ginsenosides level, high levels of glucose residues, and a higher content of di- and trisaccharides, exhibited a stronger activity in ameliorating fat accumulation and metabolic and gut microbiota dysregulation. In particular, white ginseng restored the phyla Firmicutes/Bacteroidetes ratio [16]. The prebiotic effect of ginseng polysaccharides has been investigated to elucidate the biotransformation and consequent biological activities of ginsenosides under different physio-pathological conditions. The ginsenosides, together with saccharides, positively regulate gut microbiota by acting as an energy substrate for specific intestinal bacteria. Moreover, they are able to reshape the gut microbial environment, triggering several molecular and cellular signaling pathways, which determines their therapeutic effects. The exposure to ginseng polysaccharides enhanced the microbial deglycosylation and intestinal absorption of ginsenoside Rb1 [17,18]. The synergistic effect of polysaccharides and ginsenosides recovered the gut microbiota composition and increased several beneficial mucosa-associated bacterial taxa such as Clostridiales, *Bifidobacterium*, and Lachnospiraceae, but decreased harmful bacteria *Escherichia*-*Shigella* and Peptococcaceae. This association may be used as immunostimulants targeting the microbiome–metabolomics axis under immunosuppressive and intestinal damage conditions [19,20]. Overall, the analyzed data suggest that the effect of the ginseng phytocomplex is significantly related to the activity on and of the intestinal microbiota.

#### 3.1.3. Chitosan

Chitosan and chitosan oligosaccharide (COS) are two derivates of the polysaccharide chitin, a common component of exoskeletons of arthropods and insects and a component of fungi cell walls. Chitosan is a deacetylated polymer of N-acetyl glucosamine derived from chitin. COS is an oligomer of β-(1→4)-linked d-glucosamine and represents the most studied and easily obtained derivate from chitin. COS is widely water soluble and readily absorbed through the human intestine, so it has been recently studied as an enteric coating for cells, drug and DNA delivery enhancement, and as a nutraceutical. In vivo, chitosan has shown lipid-binding properties, thus reducing their gastrointestinal uptake, and lowering serum cholesterol. Indeed, COS and its derivatives have shown interesting biological activities interacting with many pathways, thus inhibiting nuclear factor kappa B (NF-κB) and mitogen-activated protein kinase (MAPK) pathways, and the activation of AMP-activated protein kinase (AMPK) [21,22]. Whether these beneficial properties of chitosan and COS are obtained through a variation of microbiota is still debated. This observation was partially confirmed in human trials: a meta-analysis study on overweight or obese adults suggested a significant weight loss and a decrease in total cholesterol levels following chitosan treatment compared to placebo [23]. Another study, involving 10 healthy volunteers after 4 weeks of chitosan supplementation, displayed considerable variations in the composition of microbial patterns among different persons, highlighting the high complexity and individuality for each subject. A raised level of fecal *Bacteroides* spp. in response to chitosan intake was found in all samples, while the *Bifidobacterium* spp. level increased or was unchanged [24]. A clinical trial performed considering COS supplementation in Chinese coronary heart disease patients [25] highlighted that 1–2 g/day of COS consumption for 6 months decreased the number of *Faecalibacterium*, *Alistipes*, and *Escherichia* spp. and significantly increased the *Bacteroides*, *Megasphaera*, *Roseburia*, and *Prevotella* populations and *Bifidobacterium* spp. Additionally, COS consumption increased *Lactobacillus*, *Lactococcus*, and *Phascolarctobacterium* spp., which have been reported to be associated with antioxidant properties and lipid balance [26]. A chitosan derivate, carboxymethyl chitosan, also showed an antibiotic effect in a mouse model: a colon microbiota analysis after treatment showed a significant decrease in the OTU number and relative species abundance, with a severe disbalance caused by a rise in Enterobacteriaceae, Lachnospiraceae, and several other bacteria. These variations were observed alongside a worsening of glucose and lipids homeostasis [27]. An exploratory study on the fermentation of COS in vitro and COS supplementation in a mouse model indicated that COS reduced genus *Lactobacillus* and *Bifidobacterium* and impaired *Desulfovibrio*, with an increase in the *Akkermansia* population [28]; the same increase in *Akkermansia* spp. was observed after COS treatment in a model of diabetic mice [29]. COS showed a protective effect against colorectal cancer (CRC) in a model of obese mice by reversing the imbalance of bacteria and fungi, specifically, by reducing the abundance of *Escherichia-Shigella*, *Enterococcus*, and *Turicibacter* spp., and increasing the levels of *Akkermansia* spp. as well as butyrate-producing bacteria and *Cladosporium* spp. [30]. The increase in *Akermansia muciniphila* corroborated the effect observed in HFD-fed mice where two kinds of low-molecular-weight chitosan oligosaccharides (LMW) significantly decreased inflammatory bacteria such as *Erysipelatoclostridium* and *Alistipes* spp., while *Akkermansia* and Gammaproteobacteria, which are considered beneficial, increased significantly [31]. An in vitro study on human colon cells showed how chitosan derivatives with a high number of deacetylated units impaired many bacterial populations, such as *Bifidobacterium* spp., *Eubacterium rectale*/*Clostridium coccoides*, *C. Histolyticum*, and *Bacteroides/Prevotella* spp. [32]. The large number of studies on different models highlighted a beneficial impact of *Akkermansia muciniphila* treatment on obesity. *A. muciniphila* has been characterized as a beneficial player in the body metabolism, and it has been proposed for the treatment of metabolic disorders associated with obesity, as well as being considered among next-generation therapeutic agents.

#### 3.1.4. β-Glucans

β-glucans are glucose polymers present in the cell walls of fungi, yeast, and cereals. They are widely found in common foods, such as oats, barley, sea weeds, and mushrooms. Several properties have been attributed to beta-glucans, such as anticancer, antidiabetic, anti-inflammatory, and immune-modulating effects [33,34,35]. The prebiotic effect of β-glucans was demonstrated in several clinical trials on 52 healthy subjects. Indeed, a β glucan-enriched diet was able to increase the levels of *Roseburia hominis*, *Clostridiaceae* (*Clostridium orbiscindens* and *Clostridium* spp.), and *Ruminococcus* spp. and to reduce the levels of Firmicutes and Fusobacteria. The results also showed a marked increase in levels of the main SCFAs, such as 2-methyl-propanoic, acetic, butyric, and propionic acids [36]. Furthermore, many in vivo and in vitro studies have shown that β-glucans have a significant prebiotic effect due to their ability to modulate gut microbiota; in particular, the consumption of β-glucans was associated with a growth of lactobacilli and Biphobacteria and with a significant increase in the levels of SCFA, which is useful for gut integrity and functionality. The results of the mouse model also suggested that high doses are more effective than lower ones and that oats-derived β-glucans are more effective in comparison to barley β-glucans [37,38,39]. This evidence suggests that β-glucans have a positive impact on gut microbiota and improve the lipid and glycaemic profile, proposing an excellent adjuvant to hypocaloric diets [40].

#### 3.1.5. Psyllium

Psyllium is a water-soluble fiber, derived from the husks of seeds of *Plantago ovata* Forsk.fr, and able to form a viscous gel. It is used for managing intestinal regularity; reducing appetite; and interfering with the absorption of carbohydrates, lipids, and bile salt [2]. Its consumption in healthy subjects and patients with constipation highlighted small but significant effects on the microbiota; in healthy subjects, it causes an increase in *Veillonella* and a decrease in *Subdoligranulum* spp., while in constipated patients, an increase in *Lachnospira*, *Faecalibacterium*, *Phascolarctobacterium*, *Veillonella*, and *Sutterella* spp. and a decrease in Coriobacteria and *Christensenella* spp. have been observed, related to the modification of the fecal levels of acetate and propionate [41]. Further studies confirm the benefit of psyllium on the frequency of the hive, on the microbiota level, and on the concentration of SCFA [42]. By evaluating the impact on bifidobacteria, it was shown that the administration of psyllium in healthy women significantly affects the levels of these bacteria, despite the increase in the overall fecal bacterial burden. In vitro investigations have clarified how the psyllium fiber exerts prebiotic effects on bifidobacteria through partial hydrolysis, exhibiting effective bifidogenic activity only in the case of low levels of fecal bifidobacteria before treatment [43].

#### 3.1.6. Guar Gum

Guar gum is a fiber derived from the seed of Indian leguminous plant *Cyamopsis tetragonolobus* (L.) Taub. Chemically, it is a polymer of D-galactose and D-mannose called galactomannan [2]. Guar gum intake in healthy volunteers was correlated with an increase in *Ruminococcus, Fusicatenibacter, Faecalibacterium*, and *Bacteroides* spp. and a reduction in *Roseburia, Lachnospiracea*, and *Blautia* spp., associated with an improvement in defecation frequency; stool consistency; and the abundance of metabolites, including butyrate, acetate, and amino acids [44]. Data were partially confirmed by evaluations of a larger scale of samples, where in one case, the consumption of guar gum enhanced the increase in *Bifidobacterium, Ruminococcus*, and *Megasphaera* spp. and inhibited *Bacteroides* and *Phascolarctobacterium* spp. growth. This effect influenced the stool consistency without altering the frequency of the hive [45]. Furthermore, the administration of a guar gum-enriched diet has shown noteworthy effects: a transitory ability to favor the development of bifidobacteria [46]. In particular, effects of considerable interest were highlighted. The administration of guar gum in 13 children suffering from autism spectrum disorder, with concomitant dysbiosis and constipation, was able to modify the relative abundance of genus *Blautia* and increased *Acidaminococcus* spp., also reducing genera *Streptococcus, Odoribacter* and *Eubacterium* [47]. Consumption in 15 hemodialysis patients with concomitant constipation was correlated with an increase in bifidobacteria, *Bacteroides* spp., and lactobacilli and a reduction in the *Clostridium* XVIII cluster [48].

#### 3.1.7. Inulin

Inulin is a polysaccharide extracted principally from chicory and produced by many plants. From a chemical point of view, it belongs to the inulin-type fructans family (ITFs), which covers β-(2←1) linear fructose polymers, such as oligofructose and fructo-olygosaccharides (FOS), as well as inulin itself [2]. In a clinical study including 44 healthy adults with mild constipation, the intake of inulin 12 g/day was correlated with specific changes in the relative amount of *Anaerostipes*, *Bilophila*, and *Bifidobacterium* spp., associated with gut function improvement, in the absence of significant differences in the uniformity or diversity of the microbiota [49]. Results were partially confirmed by a subsequent evaluation carried out on 26 healthy individuals, where the administration of 9 g/die of inulin was associated with a greater proportion of the genus *Bifidobacterium*, a reduced level of non-classified Clostridiales, and the decreasing tendency of Oxalobacteraceae. Microbial effects were accompanied by greater satiety and a reduced desire to eat sweet, salty, and fatty foods; these changes were transient and tended to disappear 3 weeks after the cessation of administration [50]. The prebiotic effect of inulin depends on the high or low consumption of fiber. Subjects with a higher consumption of fiber showed an increase in *Bifidobacterium* and *Faecalibacterium* spp. and a decrease in *Coprococcus*, *Dorea*, and *Ruminococcus* spp., whereas in subjects with a reduced intake of fiber, an increase in *Bifidobacterium* spp. occurred, highlighting that subjects with a regular intake of fiber experience the greatest benefit from the prebiotic effect of inulin [51]. These effects could be advantageous in the case of overweight and obesity: the administration of 16 g/day of inulin plus oligofructose in obese women resulted in an increase in *Bifidobacterium* asp. and *Faecalibacterium prausnitzii* contextually to a reduction in *Bacteroides intestinalis*, *Bacteroides vulgatus*, and *Propionibacterium* spp. Moreover, a weak decrease in the fat mass and plasma levels of lactate and phosphatidylcholine was observed [52]. A similar evaluation in 12 overweight adults showed that the administration of 20 g/day of inulin-propionate ester caused changes at the class level, with an increase in Actinobacteria and a decrease in Clostridia at the order level, and a decrease in the proportion of Clostridiales, and at the species level, greater proportions of *Anaerostipes hadrus*, *Bifidobacterium faecale*, *Bacteroides caccae*, *Bacteroides uniformis*, *Bacteroides xylanisolvens*, and *Fusicatenibacter saccharivorans* and a lower percentage of *Blautia obeumin*, *Blautia lutea, Bacillius fluminis, Blautia obeum*, *Eubacterium ruminantium, Anaerostipes hadrus*, and *Prevotella copri* were detected [53]. An investigation in 12-year-old overweight subjects also revealed significant changes in the microbiota with an increase in the species of the genus *Bifidobacterium* and a decrease in *Bacteroides vulgatus* and *Faecalibacterium prausnitzii* [54]. The use of inulin and similar fructans proved to be of considerable interest due to the possibility of intervention at the microbiota level, in the management of gastroenterological disorders that are likely associated with both overweight and obesity.

### 3.2. Food Supplements with the Main Goal of Appetite Regulation

#### 3.2.1. Whey Protein

Whey protein (WP), due to its high content in branched chain amino acids, can stimulate a higher level of muscle protein synthesis than other proteins, such as casein and soy [2,55]. For this reason, the consumption or supplementation of WP is used to support the gain of muscle mass and to optimize body composition in athletes. In addition, WP exerts several health benefits, and it could represent a valuable tool against obesity, modulating appetite and the plasma levels of satiety hormones, such as insulin, ghrelin, cholecystokinin, and glucagon-like peptide 1 [56]. In a randomized pilot study in cross-country runners whose diets were supplemented with WP (20 g isolated WP + 10 g hydrolyzed WP) for 10 weeks, the increased abundance of Bacteroidetes phylum and the reduced presence of health-related taxa, including *Roseburia, Blautia* spp., and *Bifidobacterium longum*, were reported. These results suggested that long-term WP supplementation may negatively affect gut microbiota [57]. In contrast, an in vivo study showed that the 10-week supplementation of WP in 5-week-old mice fed with a HFD was able to reduce weight gain and modulate gut microbiota by increasing the portion of murine lactobacilli. These changes were detectable only in mice that started dietary intervention after 5 weeks, without prior consumption of WP, thus suggesting that the effect of whey protein on the body composition and gut microbiota of mice depends on diet duration and stage of life during which the diet is provided [58]. Another study conducted on a mouse model showed that the 21-week treatment of WP in HFD-fed mice could increase the portion of Lactobacillaceae/*Lactobacillus* and decrease Clostridiaceae/*Clostridium* [56]. Furthermore, in vitro studies performed using a gastrointestinal digestion model, showed that WP can lead to an increase in Bifidobacteriaceae and Lactobacillaceae, and *Bifidobacterium* and *Lactobacillus* spp. [59]. The current evidence shows controversial effects exercised by WP on gut microbiota, suggesting that further studies on the putative prebiotic action of WP are needed.

#### 3.2.2. Chlorogenic Acid

Chlorogenic acid (CGA), one of the most common dietary polyphenols, is a major component of coffee and some other plant species. CGA shares antioxidant and anti-inflammatory capabilities with other polyphenols; however, other properties have recently been reported in basic and clinical research studies, with alleged reduced risk of a variety of diseases [2]. Mainly based on animal studies, it has been observed that CGA exerts pivotal roles on glucose and lipid metabolism regulation, which has an impact on related disorders such as diabetes and obesity, with consequent reduced risks of cardiovascular diseases, cancer, and fatty liver disease [60]. The contribution of microbiota to these effects has been alleged in many studies, but no microbiota variation pattern following CGA supplementation has been identified. A pilot clinical trial conducted on 26 patients with diabetes and non-alcoholic fatty liver disease (NAFLD) indicated a significant decrease in body weight after the consumption of 200 mg caffeine plus 200 mg chlorogenic acid supplementation for 3 months in comparison to patients who consumed only chlorogenic acid or caffeine supplementation [61]. As noted in preclinical studies, gut Bifidobacteria increased in the caffeine plus chlorogenic acid group; however, there were no statistically significant differences within and between the groups in any of the bacteria numbers. Instead, in vivo studies showed contrasting results; CGA supplementation in obese mice models inhibited the growth of *Desulfovibrionaceae, Ruminococcaceae*, *Lachnospiraceae*, *Erysipelotrichaceae* and *Oscillospiraceae* genera and increased the growth of *Bacteroidaceae* and Lactobacillaceae [62,63], but CGA was also able to inhibit bacteria belonging to the genera *Blautia*, *Sutterella*, and *Akkermansia* and increase butyric acid levels, mainly due to *Ruminococcus* [64]. Similar to observations in other studies, an increase in the population of *Lactobacillus* and *Bifidobacterium* spp. and a decrease in the population of *Escherichia coli* were observed in an animal model fed with a CGA-enriched diet; this effect was proportional to the CGA supplementation dose [65]. In vitro studies conducted on human colon cells showed how the fermentation of coffee polyphenols such as CGA could lead to the proliferation of bifidobacteria [66,67]. Several functions have been attributed to bifidobacterial presence after CGA supplementation, such as the lysis of indigestible carbohydrates, defense from pathogens, vitamin B production, antioxidation, and immune system stimulation. Furthermore, reciprocal interactions between bifidobacteria and butyrate-producing colon bacteria, mainly *Faecalibacterium prausnitzii* and *Roseburia* spp., are suggested [68]. These interactions can favor the co-existence of bifidobacterial strains with butyrate-producing colon bacteria in the human colon, finally resulting in SCFA production, which can beneficially modulate adipose tissue, skeletal muscle, and liver tissue function, and improve glucose homeostasis and insulin sensitivity [69].

### 3.3. Food Supplements with the Main Goal of Energy Expenditure Modulation

#### 3.3.1. Curcumin

Curcumin is a natural phenolic component derived from *Curcuma longa* L. It can be used in several different fields, such as the food, textiles, and pharmaceutical industries. Although curcumin has a wide range of therapeutic impacts, it shows extremely low bioavailability, which requires the use of pharmaceutical technologies to increase its bioavailability. There is abundant evidence on the beneficial use of curcumin in several conditions, such as cancer, diabetes, autoimmune diseases, and neurodegenerative diseases [2,70,71,72]. Many studies have shown that curcumin is able to positively modify gut microbiota composition. A human randomized placebo-controlled trial showed that curcumin oral supplementation (curcumin 1000 mg plus piperine extract 1.25 mg every tablet—3 times/day) significantly changed the gut microbiota composition differently in subjects, with a significantly higher overall reduction in bacterial species than the placebo group. Although the modifications varied individually, it was found that curcumin supplementation generally promoted an increase in *Clostridium*, *Bacteroides*., *Citrobacter, Cronobacter*, *Enterobacter*, *Enterococcus*, *Klebsiella*, *Parabacteroides*, and *Pseudomonas* spp. and decreased the abundance of *Blautia* and *Ruminococcus* spp. [73]. In HFD-fed rats, curcumin significantly altered the gut microbiota composition, counteracting the HFD-induced abundance of *Ruminococcus* spp., which is described as being associated with diabetes and inflammation [74]. In addition, in rats, curcumin supplementation for 15 days (100 mg/kg) decreased the abundance of Prevotellaceae, Bacteroidaceae, and Rikenellaceae, which are families associated with the onset of systemic diseases, and increased the amount of bifidobacteria and lactobacilli, which have been shown to possess antitumoral functions [75,76,77]. Moreover, in a colorectal cancer mouse model, curcumin treatment decreased the microbial concentration of *Prevotella* and *Ruminococcus* spp., a cancer-related species [73,78]. The current evidence suggests a protective effect of curcumin due to its ability to generate a shift from pathogenic to beneficial bacteria strains in the gut.

#### 3.3.2. L-Carnitine

L-Carnitine (L-C) is an amino-acid derivative produced from the amino-acids lysine and methionine and is widely present in animal tissues [2]. Numerous studies have taken into consideration the role of L-Carnitine in the elevation of endogenous levels of trimethylamine-N-oxide (TMAO), a factor correlated with a significant increase in cardiovascular risk. Studies in human and animal models have suggested that several bacteria, such as Deferribacteraceae, Enterobacteriaceae, Anaeroplasmataceae, Prevotellaceae, Ruminococcaceae, and Lachnospiraceae, are engaged in TMAO production or the TMAO level [79,80,81,82,83,84,85], including the presence of specific genera, such as *Mitsuokella*, *Fusobacterium*, *Desulfovibrio*, and *Methanobrevibacter smithii* [85]. Evaluating the phenomenon from the point of view of enterotypes, the enterotype characterized by *Prevotella* was associated with higher plasma levels of TMAO than the *Bacteroides* enterotype [86]. In some studies, the *Emergencia timonensis* species has been identified as the main actor of the transformation of γ-butyrobetaine to TMAO; however, its presence was not highlighted as the prevalent causal element of this process in a study performed in elderly women, confirming the role also played by other described bacteria [87]. On the other hand, L-Carnitine supplementation provided a beneficial effect in patients undergoing hemodialysis, where it improved muscle discomfort, gastrointestinal disorders, and microbiota with a decrease in the abundance of genus *Clostridium* subcluster 4 [88]. A further evaluation of a similar model reported that the oral supplementation of L-Carnitine was associated with increased TMAO levels and might be ascribed partially to its inhibitory actions on glycation end products (AGE) [89]. In a mouse model, a high intake of L-carnitine induced an increase in Coriobacteriaceae, *Anaerobiospirillum* spp., *Akkermansia_muciniphila* and *Helicobacter pylori*, resulting in an increased TMAO metabolism [90]. The interaction between L-Carnitine and microbiota in most studies has been investigated as a fundamental element of the negative effects on human health related to the production of TMAO. However, in particular clinical conditions, such as those related to hemodialysis, it could have positive effects. Future studies will be necessary to definitively clarify any fields of application and real potential.

### 3.4. Food Supplements with the Main Goal of Fat Metabolism

#### 3.4.1. Resveratrol

Resveratrol (RSV) is a polyphenolic compound belonging to the stilbenoid family; it is widely found in its trans-isomer form in various plants, such as *Polygonum cuspidatum* Siebold & Zucc.; fruits, including grapes and berries, peanuts, and red wine. Due to the extensive glucuronidation in the liver and intestine and sulfation in the liver, its bioavailability is very low [2,91]. The effects of RSV supplementation have been studied in both animals and clinical trials. Several health benefits seem to be associated with RSV, including the prevention of cancer [92], obesity, and type 2 diabetes [93]; antiaging effects [94]; and the promotion of cardiovascular health [95]. A randomized, double-blind, placebo-controlled trial demonstrated that the supplementation of epigallocatechin-3-gallate and RSV (282 and 80 mg/day, respectively, for 12 weeks) reduced the abundance of Bacteroidetes phylum in men but not in women, increasing fat oxidation and skeletal muscle mitochondrial oxidative capacity [96]. Recent in vivo studies have shown that RSV induces changes in gut microbiota, which could lead to lower body weight and body fat and improve glucose homeostasis and obesity-related parameters [93]. In mice, high intakes (200 mg/kg/day) of RSV provided an increase in *Lactobacillius* and *Bifidobacterium* spp. [97]. Meanwhile, RSV decreased TMAO levels by inhibiting commensal microbial trimethylamine (TMA) production via gut microbiota remodeling in ApoE−/− mice, thus reducing arteriosclerosis risk [98]. Furthermore, RSV supplementation (450 mg/kg/day for 2 weeks) resulted in a reduction in the Bacteriodetes/Firmicutes ratio and *Lachnospiraceae* and an increase in *Parabacteroides*, *Bilophila*, and *Akkermansia* spp. in HFD-fed mice, improving skeletal muscle insulin sensitivity, glucose utilization, and metabolic rate [99]. Conversely, the Firmicutes/Proteobacteria ratio was increased in hypertensive rats supplemented for 90 days with a drink containing 50 mg/L of resveratrol, restoring systolic and diastolic blood pressure [100]. Other variations in the gut microbiota have been observed following RSV and the supplementation of some of its active metabolites, such as a reduction in *Lactococcus*, *Clostridium, Oscillibacter, Hydrogenoanaerobacterium* spp. (200 mg/kg/day for 8 weeks) [101], *Parabacteroides jonsonii* DMS 18315, *Alistipes putredinis* DMS 17216, *Bacteroides vulgatus* ATCC 8482 (60 mg/kg/day for 5 weeks), and *Enterococcus faecalis* (200 mg/kg/day for 12 weeks), resulting in improved body weight and glucose and lipid profiles [97,102]. Piceatannol, derived from the CYP450 metabolism of RSV, shows similar effects on gut microbiota to those described above in mouse models [103]. Based on the evidence, RSV can lead to health benefits modulating gut microbiota.

#### 3.4.2. Flaxseed

*Linum usitatissimum* L. is a widely known medicinal plant worldwide, as well as a textile fiber source and a dietary source. For medical applications, the part of interest of the plant is represented by the seed (semen) (flaxseed), which is rich in oil (consisting of polyunsaturated fatty acids (PUFAs), particularly w-3); lignans such as linustatin, neolinustatin, and linamarin; and soluble and insoluble complex polysaccharides (EMA assessment). According to the WHO and EMA monographs, flaxseed is used mainly for the treatment of constipation and gastrointestinal discomfort relief [2,104]. Flaxseed has been investigated in weight loss and some clinical trials have reported a beneficial effect of its oil and milled seed. The daily consumption of 30 g of brown milled flaxseed associated with lifestyle intervention was found to be more effective than only lifestyle changes in reducing body weight and metabolic markers in patients with metabolic syndrome [105]. The prebiotic effect of flaxseed constituents has been considered and deeply investigated. A high-quality clinical trial showed the importance of gut metabolization on the biological effect of flaxseed in obesity [106]. Fifty-eight obese post-menopausal women were enrolled and divided into three groups in a 6-week trial with parallel group intervention, which was single-blinded: *Lactobacillus paracasei* F19, flaxseed mucilage (10 g), or placebo groups were considered. Flaxseed intake over 6 weeks, to a higher extent compared to the placebo, improved insulin sensitivity in terms of serum C-peptide reduction and enhanced insulin response. Lipid metabolism and inflammatory markers were decreased after a 6-week intervention with flaxseed, but differences compared to placebo groups were not significant. The probiotic supplementation did not modify considered parameters. Microbiota structure comparison at time zero and after intervention showed that the placebo and *L. paracasei* only exerted slight alterations in the fecal abundance of bacterial strains. In contrast, flaxseed mucilage produced a marked change in the stool abundance of bacterial genes belonging to thirty-three gut bacterial species; twenty-four decreased species were identified, such as the *Faecalibacterium* genus, and eight increased, such as the *Clostridium* genus. Authors concluded that the effects on microbiota alterations and insulin sensitivity exerted by flaxseed were not likely to be interconnected. In vivo studies confirmed the prebiotic effect of soluble polysaccharides of *L. usitatissimum* seed and, interestingly, the effect on the restoration of SCFA production (such as propionic and butyric acid) by intestinal bacteria in a population of high-fat diet-fed mice [107]. Experiments in animal models also showed that flaxseed supplementation reduced the *Akkermansia muciniphila* abundance provoked by linseed inclusion in the diet [108,109]. Gut microbial metabolization has been described for flaxseed fiber but also for other compounds, such as flaxseed fatty oil and lignans. In an analysis of the fecal material of subjects who took flaxseed, the presence of substances such as enterolactone, enterodiol, and matairesinol was found, which were identifiable as products of bacterial metabolism [110,111]. Enterolactone and enterodiol have been considered as the result of the microbial degradation of secoisolariciresinol diglucoside, likely conducted by *Clostridium saccharogumia*, *Eggerthella lenta*, *Blautia producta*, and *Lactonifactor longoviformis* [112]. The metabolization of secoisolariciresinol diglucoside was also associated with the presence of some *Bacteroides* spp. [113] and *Bifidobacterium* spp. [114]. The colonic complex pathway of lignan metabolization was accurately reviewed by Thumann et al. [108]. As regards flaxseed oil, an in vivo study conducted on mice observed levels of gut metabolites derived from a supplementation with sunflower oil rich in ω-6 and flaxseed oil rich in ω-3; the conjugated linolenic acid level was higher after flaxseed oil supplementation [115].

**Table 1:** Summary of clinical trials reporting prebiotic effects of these natural substances and their impact on gut microflora.

Substance	Subjects	Intervention	Clinical Outcome	Gut Microflora Modifications
**Substances with Evidence of Weight Loss Associated with Modifications of the Microbiota**				
**Chlorogenic Acid [61]**	26 patients with diabetes and non-alcoholic fatty liver disease (NAFLD)	3 months of 200 mg caffeine plus 200 mg chlorogenic acid supplementation	Significant decrease in body weight	Non-significative bifidobacteria increase in the caffeine plus chlorogenic acid group
**Inulin [49,50,51,52,53,54]**	44 healthy adults with mild constipation	12 g/day inulin intake	Gut function improvement	Changes in relative abundances of *Anaerostipes*, *Bilophila*, and *Bifidobacterium* spp.
	26 healthy individuals	9 g/die inulin intake	Greater satiety	Greater proportions of the genus *Bifidobacterium*, a reduced level of non-classified Clostridiales. and a tendency to decrease Oxalobacteraceae
	30 obese women	Inulin/oligofructose 50/50 mix 16 g/day for 3 months	Slight decrease in fat mass and plasma levels of lactate and phosphatidylcholine	*Bifidobacterium* spp. and *Faecalibacterium prausnitzii* increase and *Bacteroides intestinalis, Bacteroides vulgatus.* and *Propionibacterium* spp. reduction
	12 overweight adults	20 g/day of inulin-propionate ester		Increase in Actinobacteria, decrease in Clostridia; decrease in the proportion of Clostridiales order. *Anaerostipes hadrus*, *Bifidobacterium faecale. Bacteroides caccae*, *Bacteroides uniformis*, *Bacteroides xylanisolvens*, and *Fusicatenibacter saccharivorans* higher percentage and a lower percentage of *Blautia obeumin*, *Blautia lutea* and *Bacillius fluminis*, *Blautia obeum*, *Eubacterium ruminantium*, *Anaerostipes hadrus*, and *Prevotella copri*
	42 12-year-old overweight subjects	8 g/day of oligofructose enriched inulin for 16 weeks	Significant decreases in body weight and serum triglycerides	*Bifidobacterium* spp. increase and decrease in *Bacteroides vulgatus* and *Faecalibacterium prausnitzii*
***P. ginseng* [15]**	10 obese women	8 g dry extract for 8 weeks	Weight loss effect and slight modifications of gut microbiota	In effective weight loss group, change in levels of *Blautia, Faecalibacterium* spp. In ineffective weight loss group, change in levels of *Bifidobacterium*, *Blautia*, and *Clostridium* at the genus level
**Substances with Evidence of Metabolic Modifications Potentially Favorable to Weight Loss Associated with Modifications of the Microbiota**				
**Resveratrol (RSV) [96]**	37 overweight and obese men and women	Supplementation of epigallocatechin-3-gallate and RSV (282 and 80 mg/day, respectively, for 12 weeks)	Increased fat oxidation and skeletal muscle mitochondrial oxidative capacity	Reduced abundance of Bacteroidetes phylum in men but not in women
**Flaxseed [106]**	58 obese postmenopausal women	Flaxseed mucilage (10 g) for 6 weeks	Improved insulin sensitivity related to the decrease in the serum C-peptide and insulin response	24 decreased species *in Faecalibacterium* genus and 8 increased species in *Clostridium* genus
**Substances with Evidence of Changes in the Microbiota Associated with Other Effects (Which in the Animal Model are Associated with Weight Loss)**				
**β-glucans [36]**	26 healthy subjects	2 months of 3 g/day of barley β-glucans	Marked increase in levels of the main SCFA	Increased levels of *Roseburia hominis*, Clostridiaceae (*Clostridium orbiscindens* and other *Clostridium* spp.), *Ruminococcus* spp. and reduced abundance of Firmicutes and Fusobacteria
**Chitosan and COS [24,26]**	10 healthy volunteers	3 g chitosan/day before meal for 28-day supplement period		Increased level of *Bacteroides* spp.
	120 Chinese coronary heart disease patients	COS consumption of 1-2 g/day for 6 months	Improved blood urea nitrogen and serum creatinine. Higher circulating antioxidant levels. Increased SOD and GSH serum levels. Reduced levels of ALT and AST. Improved lipid profiles	Decreased *Faecalibacterium, Alistipes*, and *Escherichia* spp. abundance. *Bacteroides*, *Megasphaera*, *Roseburia*, *Prevotella*, and *Bifidobac-terium* spp., increased abundance of *Lactobacillus*, *Lactococcus*, and *Phascolarctobacterium* spp.
**Curcumin [73]**	30 healthy subjects (14 analyzed)	1000 mg curcumin plus 1.25 mg extract of piperine every tablet—3 times/day		Increase in *Clostridium*, *Bacteroides.*, *Citrobacter*, *Cronobacter*, *Enterobacter*, *Enterococcus*, *Klebsiella*, *Parabacteroides*, and *Pseudomonas* spp. and decreased abundance of *Blautia* and *Ruminococcus* spp.
**Green Tea [5]**	Healthy subjects: 8 males, 4 females	400 mL per day for two weeks	Elevation in SCFA, and reduction in bacterial LPS synthesis in feces	Increased Firmicutes to Bacteroidetes ratio, reduced fecal levels of *Fusobacterium* spp.
**Guar Gum [44,45,46,47,48]**	20 healthy volunteers	5 g of guar gum three times/day for 3 weeks	Improvement in defecation frequency; stool consistency; and abundance of butyrate, acetate, and amino acids	Increase in *Ruminococcus*, *Fusicatenibacter*, *Faecalibacterium*, and *Bacteroides* spp. and a reduction in *Roseburia*, *Lachnospiracea*, and *Blautia* spp.
	44 healthy volunteers	5 g/day guar gum for 3 months	Altered stool consistency	*Bifidobacterium*, *Ruminococcus*, and *Megasphaera* spp. increase and *Bacteroides* and *Phascolarctobacterium* spp. inhibited growth
	31 healthy volunteers	3.4 g/day guar gum for 21 days		Transitory bifidobacterial increase
	13 children suffering from autism spectrum disorder with concomitant dysbiosis and constipation	6 g/day guar gum	Increased defecation frequency and reduced irritability	Increased *Acidaminococcus* spp. and reduced genera *Streptococcus*, *Odoribacter*, and *Eubacterium* spp.
	15 hemodialysis patients with concomitant constipation	5.1 g/day guar gum for 4 weeks	Improved the individual stool form and decreased the constipation	Increase in bifidobacteria, *Bacteroides* spp. and lactobacilli and reduction in the *Clostridium* XVIII cluster
**L-carnitine [88]**	15 Japanese patients receiving hemodialysis	L-carnitine tablets (900 mg) for 3 months	Improved muscle discomfort, gastrointestinal disorders	Decrease in the abundance of genus *Clostridia* subcluster 4
**Whey Proteins [57]**	24 cross-country runners	10 weeks 20 g isolated WP + 10 g hydrolyzed WP		Increased abundance of Bacteroidetes phylum; decreased presence of health-related taxa, including *Roseburia*, *Blautia* spp., and *Bifidobacterium longum*
**Psyllium [41]**	8 healthy volunteers and 16 constipated patients	7 days of 7 g/day psyllium	Increased acetate, propionate, and butyrate, correlated with increased fecal water	Healthy adults increased *Veillonella* and decreased *Subdoligranulum* spp. In constipated subjects, increased levels of *Lachnospira*, *Faecalibacterium*, *Phascolarctobacterium*, *Veillonella*, and *Sutterella* spp., decreased uncultured Coriobacteria and *Christensenella* spp.

## 4. Discussion

The evaluation of the effectiveness and related biological mechanisms of natural products mainly used in the management of overweight depicts different targets and underlined mechanisms, including carbohydrate and fat metabolism and/or increased energy expenditure. Interestingly, current evidence suggests that the modulation of gut microflora provided by some nutraceuticals and herbal food supplements may play a relevant role in intestinal homeostasis. The analysis performed in this review underlines the effect on overweight and related pathological conditions exerted by nutraceuticals and herbal food supplements intervening in the gut microbiota structure and their reshaping abilities. In particular, this work highlights the complexity of specific modifications of the microbial environment produced by natural products. It is worth noting that in a recent review, which comprehensively took into account the most used food supplements for the management of overweight [2], all natural products with a demonstrated prebiotic effect were included. In vivo and in vitro studies have evidenced a prebiotic effect of top selling food supplements, such as white kidney bean, glucomannan, agar, caralluma, spirulina, coffee/caffeine/guaranà, bitter orange, capsaicin, capsaicinoids and capsinoids, pyruvate, dyacilglycerol, liquorice, conjugated linoleic acid, aloe vera, grapefruit, mangosteen, chromium and lipoic acid, whereas more sound data could be retrieved for some natural products investigated in clinical trials. This review depicts the up-to-date state of the art of the fascinating complexity of gut microbiota modulation exerted by nutraceuticals and herbal food supplements investigated in clinical trials. Some of these substances have been shown to be able to act on *Bifidobacterium*; *Lactobacillus*; *Akkermansia*; and productive butyrate species, such as *Faecalibacterium* and *Roseburia* spp. The ability to favor the development of bifidobacteria is considerably relevant for a series of different effects, including the immuno-modulation processes and protection against pathogens; cross-talk with the butyrate producers, such as *Faecalibacterium prausnitzii* and *Roseburia* spp.; and the ability to intervene in the metabolism of macro- and micronutrients with lysis of indigestible carbohydrates, vitamin B group synthesis and antioxidants substances [68]. Furthermore, the ability to positively modulate butyrate-producing bacteria potentially confers other metabolic benefits, favoring the modulation of adipose organ function, liver and skeletal muscle function, with the amelioration of glucose levels and improved insulin sensitivity [69]. The upregulation of *Lactobacillus* spp. is a potentially favorable element in the management of various conditions, such as the health maintenance of reproductive [116] and urological [117] systems. Crovesy et al. also described numerous benefits in relation to weight control for different *Lactobacillus* spp., both taken individually and combined with other nutraceutical products. [118]. Several nutraceuticals show the ability to favor the modulation of *Akkermansia muciniphila*, a strain actively investigated in recent years. *Akkermansia muciniphila* is related to a series of extremely favorable effects in the context of weight control, reduction in body fat, and the management of many of metabolic problems. Everard et. al. reported that *Akkermansia muciniphila* can act by increasing endocannabinoids gut levels, which play a relevant role in inflammation, gut barrier and gut peptide secretion, reverting fat-mass gain, adipose tissue inflammation, insulin resistance, and metabolic endotoxemia [119]. Xu et al. proposed that *Akkermansia muciniphila* also intervenes favorably in the modulation of liver and metabolic disorders related to lipid metabolism, modifying the metabolic pathways present in an obesity condition [24]. Abuqwider et. al, starting from the analysis of 804 studies, focused on 10 randomized controlled trials that showed that *Akkermansia muciniphila* balances the dynamics of energy management, favoring a better balance of carbohydrate metabolism with a consequent reduction in low-grade inflammatory processes, and contributed to weight management and to the improvement of the metabolic parameters related to obesity [120]. *Faecalibacterium prausnitzii* and *Rosaeburia* spp. are described as being the main producer of butyrate [121], an essential short chain fatty acid for maintaining gut homeostasis and intestinal barrier function. Their presence is correlated with a better management of inflammatory processes and immune tolerance, especially in allergy-based disorders [122]. Most nutraceuticals and herbal food supplements considered in this review specifically have a prebiotic effect on these bacteria, suggesting a potential role in body weight modulation and in other conditions where butyrate production may provide an effective clinical advantage. The effects of considered nutraceuticals on selected bacterial species are summarized in Table 2.

How some nutraceuticals could exert health beneficial effects by modulating the Firmicutes/Bacteroidetes ratio, a factor of considerable interest as its alteration with the reduction in *Bacteroidetes* is related to obesity phenotypes, is yet to be elucidated [123]. Studies in human and animal models have highlighted that nutraceuticals such as β-glucans, spirulina, chlorogenic acid, resveratrol, conjugated linoleic acid, grapefruit, and mangosteen are described as being able to downgrade the Firmicutes/Bacteroidetes ratio. In the case of herbal food supplements, such as green tea and ginseng, the effects of the normalization of the Firmicutes/Bacteroidetes ratio are still debated.

## 5. Conclusions

This review shows that some natural products that are widely used as food supplements and nutraceuticals can exert a prebiotic effect contributing (1) to weight loss together with other described mechanisms, (2) to potentiating the functional effects of weight loss, and (3) to other physiological effects that are not associated with weight loss in humans. This review indicates the importance of investigating both the systemic and prebiotic mechanisms of all substances that target nutrient absorption and metabolism, suggesting that more clinical trials may increase the knowledge on this intricate topic.

## Figures and Tables

**Table 2:** Nutraceuticals that exert prebiotic effects on *Bifidobacterium* spp., *Lactobacillus* spp., *Akkermansia mucciniphila*, *Faecalibacterium prausnitzii*, and *Roseburia* spp.

	*Bifidobacterium* spp.	*Lactobacillus* spp.	*Akkermansia muciniphila*	*Faecalibacterium prausnitzii*	*Roseburia* spp.
Green Tea	X	X	X	X	X
Chitosan	X	X	X	X	X
Beta Glucans					X
Psyllium				X	
Guar Gum				X	X
Inulin				X	
Whey Protein	X	X			X
Chlorogenic Acid	X	X	X	X	X
L-Carnitine			X		
Curcumin	X				
Resveratrol	X		X		
Flaxseed	X	X		X	
